# Adapting Neutralizing Antibodies to Viral Variants by Structure‐Guided Affinity Maturation Using Phage Display Technology

**DOI:** 10.1002/gch2.202300088

**Published:** 2023-08-21

**Authors:** Frederik Peissert, Mattia Pedotti, Riccardo Corbellari, Luca Simonelli, Raoul De Gasparo, Elia Tamagnini, Louis Plüss, Abdullah Elsayed, Mattia Matasci, Roberto De Luca, Irene Cassaniti, Jose’ Camilla Sammartino, Antonio Piralla, Fausto Baldanti, Dario Neri, Luca Varani

**Affiliations:** ^1^ Philochem AG Libernstrasse 3 Otelfingen 8112 Switzerland; ^2^ Institute for Research in Biomedicine Università della Svizzera italiana (USI) Bellinzona 6500 Switzerland; ^3^ Philogen SpA Località Bellaria 35 Sovicille (SI) 53018 Italy; ^4^ Molecular Virology Unit Microbiology and Virology Department Fondazione IRCCS Policlinico San Matteo Pavia 27100 Italy; ^5^ Department of Clinical Surgical Diagnostic and Pediatric Sciences Università degli Studi di Pavia Pavia 27100 Italy

**Keywords:** adapting antibodies to viral variants, neutralizing antibodies, structure‐guided affinity maturation

## Abstract

Neutralizing monoclonal antibodies have achieved great efficacy and safety for the treatment of numerous infectious diseases. However, their neutralization potency is often rapidly lost when the target antigen mutates. Instead of isolating new antibodies each time a pathogen variant arises, it can be attractive to adapt existing antibodies, making them active against the new variant. Potential benefits of this approach include reduced development time, cost, and regulatory burden. Here a methodology is described to rapidly evolve neutralizing antibodies of proven activity, improving their function against new pathogen variants without losing efficacy against previous ones. The reported procedure is based on structure‐guided affinity maturation using combinatorial mutagenesis and phage display technology. Its use against the novel severe acute respiratory syndrome coronavirus 2 (SARS‐CoV‐2) is demonstrated, but it is suitable for any other pathogen. As proof of concept, the method is applied to CoV‐X2, a human bispecific antibody that binds with high affinity to the early SARS‐CoV‐2 variants but lost neutralization potency against Delta. Antibodies emerging from the affinity maturation selection exhibit significantly improved neutralization potency against Delta and no loss of efficacy against the other viral sequences tested. These results illustrate the potential application of structure‐guided affinity maturation in facilitating the rapid adaptation of neutralizing antibodies to pathogen variants.

## Graphical abstract

This study describes a methodology to rapidly evolve antibodies, improving their function against new pathogen variants without losing efficacy against previous ones. The procedure is based on structure‐guided affinity maturation using combinatorial mutagenesis and phage display technology; the method efficacy is demonstrated by evolving an antibody against SARS‐CoV‐2, but its use is suitable for any other pathogen.

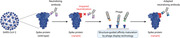

## 1 Introduction

Antibody‐based therapy has established itself as a safe and effective treatment against a plethora of diseases. The growing importance of this field is easily revealed by the annual market value of monoclonal antibodies (mAbs). From the first mAb in 1975^[^ ^1^ ^]^ to the first therapeutic successes in the 1990s,^[^ ^2^, ^3^ ^]^ the annual market value for mAbs has expanded from ≈5 billion USD in 2000 to ≈40 billion USD in 2010, 150 billion USD in 2020, and an estimated 390 billion USD in 2030.^[^ ^4^, ^5^, ^6^ ^]^ The description of technical advances that have shortened development time and decreased cost is beyond the scope of this manuscript; however, the discovery and development of therapeutic antibodies remain laborious, slow, and expensive.^[^ ^7^, ^8^ ^]^ One of the great advantages of mAbs is their remarkable specificity for their selected target (antigen). Unfortunately, this can become a major problem when the antigen mutates, since even a single amino acid substitution can have devastating effects on antibody binding and efficacy.^[^ ^9^, ^10^, ^11^ ^]^ Several pathogens have a tendency to mutate and evade the immune response, either naturally or induced by therapies. For example, antibiotic resistant bacteria are widely considered a major threat for the future.^[^ ^12^ ^]^ Similarly, viruses are known to mutate with ease, either through time (e.g., influenza, human immunodeficiency virus)^[^ ^13^, ^14^ ^]^ or geographic distribution (e.g., Dengue, Zika, and more).^[^ ^15^, ^16^ ^]^ The effect of viral escape was dramatically evident in SARS‐CoV‐2,^[^ ^17^, ^18^ ^]^ which we here use as an example to illustrate how structure‐guided affinity maturation by phage display can help adapt existing antibodies to mutating antigens rapidly and economically.

**Figure 1:**
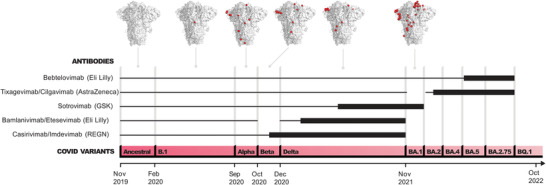
Antibody therapies were made ineffective by SARS‐CoV‐2 variants. The variants timeline is shown at the bottom. The thick black bars indicate periods when Abs were clinically available. The thick black line indicates that Abs neutralize a given variant. All Abs fail against the recent, soon to be dominant, BQ.1. Variants co‐circulation is not represented for simplicity.

Since the first reported SARS‐CoV‐2 infections in late 2019, several variants have rapidly evolved due to mutations in the viral genome (**Figure** **1**).^[^ ^19^ ^]^ Some of these mutations confer not only enhanced immune evasion properties but also increased infectivity.^[^ ^20^, ^21^, ^22^ ^]^ Highly transmissible variants such as Delta (B.1.617.2)^[^ ^20^ ^]^ and Omicron (B.1.1.529)^[^ ^21^ ^]^ have quickly spread across all continents and have replaced the wild‐type virus and other less infectious variants.^[^ ^22^ ^]^ The high intrinsic mutational rate of SARS‐CoV‐2^[^ ^23^ ^]^ combined with a selective pressure exerted by the host immune system continues to drive the evolution of SARS‐CoV‐2, and new variants will likely continue to emerge in the future, replacing currently dominant sequences.

SARS‐CoV‐2 neutralizing mAbs typically target the spike trimer, a viral glycoprotein and key mediator of viral infectivity, and its receptor binding domain (RBD) in particular.^[^ ^24^ ^]^ The spike glycoprotein mediates entry into target cells by binding to the angiotensin‐converting enzyme 2 (ACE2) receptor.^[^ ^25^ ^]^ Blocking the interaction between ACE2 and the spike trimer using mAbs, therefore, prevents virus entry into the target cells.^[^ ^25^ ^]^ A number of neutralizing mAbs have received emergency use authorization based on their clinical activity against the virus.^[^ ^26^, ^27^, ^28^, ^29^ ^]^ However, the significantly mutated spike glycoproteins of new SARS‐CoV‐2 variants, such as Delta and Omicron, have led to a substantially reduced efficacy of all approved neutralizing antibodies.^[^ ^30^, ^31^, ^32^, ^33^ ^]^ At the time of writing, no antibody authorized for human use is able to protect against the currently dominant SARS‐CoV‐2 sequences.^[^ ^34^ ^]^

One strategy to address the challenge of continuously mutating pathogens such as SARS‐CoV‐2 is the ex novo isolation of antibodies each time a novel mutant emerges. However, such isolation procedures require an extensive *in vitro* and *in vivo* characterization of the isolated clones until an antibody displaying the desired features is identified. In addition to the high cost and laborious procedures associated with this approach, the new antibody might show a reduced affinity to other viral variants or might not neutralize them at all. An attractive alternative is the adaption of existing neutralizing antibodies to viral mutants, aiming at improving binding to the new variant without losing efficacy against old ones. A fast and robust way to achieve this goal can be the structure‐guided affinity maturation of existing antibodies by combinatorial mutagenesis and phage display technology.

Phage display technology, pioneered by Sir Gregory Winter and collaborators,^[^ ^35^ ^]^ is based on the genetic integration of an antibody fragment into the pIII coat protein of filamentous phage, permitting the display of the corresponding antibody fragment on the phage surface.^[^ ^35^ ^]^ To increase the affinity of existing antibodies to a mutated target protein, phage display affinity maturation libraries can be constructed by combinatorially mutating amino acids in the complementarity‐determining regions (CDRs) of the parental antibody. The constructed libraries are subjected to an affinity capture procedure on the immobilized target antigen. This typically leads to the isolation of binding specificities exhibiting improved characteristics compared to the parental antibody. It is often advantageous to design affinity maturation libraries based on structural information of the parental antibody in complex with its cognate antigen. This is particularly relevant for the adaption of neutralizing antibodies to mutated pathogens. Careful analysis of structural data of the parental antibody bound to the target protein allows the construction of focused affinity maturation libraries, for example, targeting paratope residues close to the epitope residues mutated in the pathogen variant, thus increasing the chance of isolating high affinity antibodies adapted to the variant.

Here we describe a detailed methodology to rapidly evolve existing neutralizing antibodies of proven efficacy to recognize mutated pathogens. The protocol is based on structure‐guided affinity maturation using combinatorial mutagenesis and phage display technology. The effectiveness of the reported procedure was demonstrated by adapting the existing neutralizing antibody CoV‐X2 to the Delta variant of SARS‐CoV‐2. The affinity matured antibody, termed CoV‐X2_D, displayed sixfold improved virus neutralization against the Delta variant and no loss of efficacy against the wild‐type virus and other variants tested, compared to the parental antibody.

## 2 Results

### 2.1 Generation of Structure‐Guided Affinity Maturation Libraries

**Figure 2:**
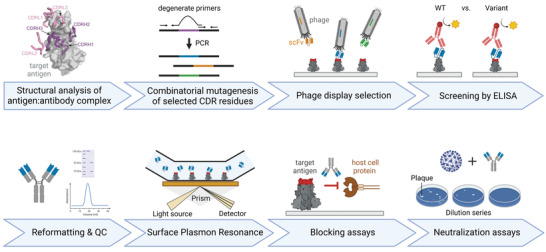
Adapting neutralizing antibodies to pathogen variants by structure‐guided affinity maturation. This figure illustrates the workflow of a structure‐guided affinity maturation procedure to improve the binding of existing neutralizing antibodies to pathogen variants. Structural analysis of the antibody in complex with its cognate antigen reveals information about which amino acids in the complementary determining regions (CDRs) of the antibody are important for interactions with the target protein. CDR residues that are in contact with amino acids mutated in the target antigen of the pathogen variant are randomized by polymerase chain reaction (PCR) using partially degenerate primers. The resulting combinatorial library is cloned into a phagemid vector, yielding an antibody phage library that is subjected to an affinity capture procedure on recombinant mutated antigen. Isolated clones are screened for binding to wild‐type and mutated antigen by enzyme‐linked immunosorbent assay (ELISA). Clones that bind to both wild‐type and mutant target protein can be characterized *in vitro* for binding kinetics (e.g., by surface plasmon resonance [SPR]), blocking assays (e.g., by ELISA), and neutralization (e.g., by plaque‐reduction neutralization assays). Figure created with BioRender. Antigen:antibody complex cartoon adapted from Barnes et al.^[^ ^25^ ^]^

**Figure 3:**
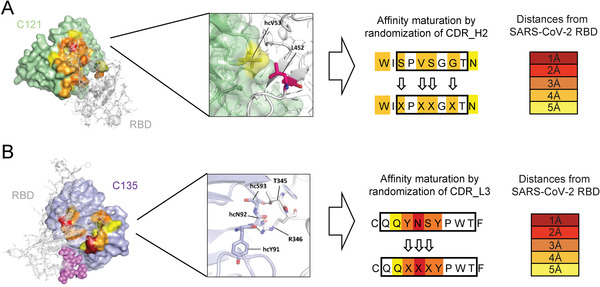
Structure‐guided design of two affinity‐maturation libraries to adapt CoV‐X2 to the Delta variant of SARS‐CoV‐2. The bispecific antibody CoV‐X2 comprises two binding specificities, termed C121 and C135. An affinity maturation library for each of the two antibodies was designed based on structural information of the antibodies bound to wild‐type spike protein obtained by Barnes et al.^[^ ^25^ ^]^ (PDB ID 7K8Q [A] and PDB ID 7K8R [B], respectively). A) Several amino acids in the CDR_H2 of C121 were predicted to be in close contact with Leu452 in the receptor binding domain (RBD) of wild‐type SARS‐CoV‐2, which is mutated to Arginine in the Delta variant. Residues S52, V54, S55, and G57 were chosen to be combinatorially mutated for the construction of the C121 affinity‐maturation library. B) Multiple amino acids in the CDR_L3 of C135 were predicted to be in close contact with Thr345 and Arg346 in the RBD of SARS‐CoV‐2. Residues Y225, N226, and S227 were selected to be combinatorially randomized to construct the C135 affinity‐maturation library.

We report a methodology for adapting existing neutralizing mAbs, making them fit to recognize emerging pathogen variants. The procedure is based on structure‐guided affinity maturation using combinatorial mutagenesis and phage display technology, as illustrated in **Figure** **2**. We demonstrate the approach here by adapting CoV‐X2, a human bispecific antibody that binds with high affinity to the early SARS‐CoV‐2 variants but lost neutralization potency against Delta.^[^ ^36^ ^]^ As a first step, both binding specificities of CoV‐X2, termed C121 and C135, were reformatted into a single chain variable fragment (scFv) format, linking the variable heavy (V_H_) and the variable light (V_L_) domains by the flexible Gly_4_SerGly_4_SerGly_4_ linker.^[^ ^37^ ^]^ The scFv fragments were cloned into the phagemid vector pHEN1 and affinity maturation libraries were constructed by combinatorial mutagenesis of selected residues. The choice of residues was based on the structural information of the two antibodies bound to the wild‐type spike glycoprotein of SARS‐CoV‐2, which had been obtained by Barnes et al.^[^ ^25^ ^]^ We focused on residues in the CDRs of the antibodies that were predicted to be in close proximity to amino acids mutated in the delta variant of SARS‐CoV‐2 (**Figure** **3**). The region defining interactive residues in the Delta variant was loosely defined to allow for structural plasticity both in the antigen and the antibody. The selected residues (S52, V54, S55, and G57 for the C121 antibody; Y225, N226, and S227 for the C135 antibody) were randomized by combinatorial mutagenesis using partially degenerate primers during polymerase chain reaction (PCR) (**Table** **1**). The transformation of the combinatorial antibody libraries into electrocompetent Escherichia coli (*E. coli)* TG‐1 cells resulted in a transformation efficiency of 4 × 10^7^ transforming units for the C121 library and 6.3 × 10^7^ transforming units for the C135 library, respectively, covering the entire theoretical diversity of each library (1.6 × 10^5^ for the C121 antibody and 8 × 10^3^ for the C135 antibody, respectively). The cloning strategy of the affinity maturation libraries is outlined in detail in Figure S1, Supporting Information.

**Table 1:** Primers for the cloning of the affinity‐maturation libraries of C121 and C135

Name	Sequence
a) LMB3long	5′ CAG GAA ACA GCT ATG ACC ATG ATT AC 3′
b) VH_C121_rev_CDR2	5′ GAA ACT TCT GTG CAT AGT TTG TMN NAC CMN NMN NAG GMN NGA TC 3′
c) VH_C121_fwd_CDR2	5′ ACA AAC TAT GCA CAG AAG TTT C 3′
d) VL_C135_rev_CDR3	5′ CTT GGC CGA ACG TCC ACG GAT AMN NMN NMN NCT GTT G 3′
e) VL_C135_fwd_CDR3	5′ TAT CCG TGGACG TTC GGC CAA G 3′
f) fdseqlong	5′ GAC GTT AGT AAA TGA ATT TTC TGT ATG AGG 3′

M: A/C; N: A/C/G/T according to IUPAC nomenclature.

### 2.2 Isolation of Affinity‐Matured Antibodies by Phage Display Technology

We subjected the generated affinity maturation libraries to an affinity capture procedure on a recombinantly produced preparation of the SARS‐CoV‐2 Delta variant RBD. The recombinant protein was expressed in mammalian cells and purified by affinity chromatography. Sodium dodecyl sulfate‐polyacrylamide gel electrophoresis (SDS‐PAGE), size‐exclusion chromatography (SEC), dynamic light scattering (DLS), and circular dichroism (CD) confirmed that the antigen was folded, monomeric, and had the expected molecular weight (Figure S2, Supporting Information). The antigen was site‐specifically biotinylated and immobilized on streptavidin coated magnetic beads. We performed one round of panning on the immobilized Delta variant RBD using the two constructed affinity maturation libraries. Isolated clones were tested for binding to recombinant wild‐type and Delta variant RBD by enzyme‐linked immunosorbent assay (ELISA). Clones yielding higher ELISA signals compared to the parental antibodies for both wild‐type and Delta variant were selected for further investigation (Figure S3, Supporting Information).

### 2.3 Antibodies Characterization

To reformat the isolated clones back into a bispecific IgG like format, we selected four C121 clones and two C135 clones based on the highest signals in ELISA. The resulting eight combinations were cloned into bispecific antibodies using CrossMab technology, expressed in Chinese hamster ovary (CHO) cells, and purified by Protein A affinity chromatography. SDS‐PAGE and size‐exclusion chromatography confirmed that all bispecific antibodies were monomeric and had the expected molecular weight (Figure S4, Supporting Information).

The binding kinetics of the novel bispecific antibodies were assessed by surface plasmon resonance (SPR). All bispecific antibodies bound at low nanomolar affinity to the RBD and S trimer of wild‐type and Delta SARS‐CoV‐2 (Tables S2 and S3, Supporting Information, **Figure** **4**, and Figure S5, Supporting Information). The best antibody candidates, CoV‐X2_D and CoV‐X2_F, were selected and further characterized, showing the same biophysical properties and quality as the parental antibody CoV‐X2 (Figure S4, Supporting Information).

**Figure 4:**
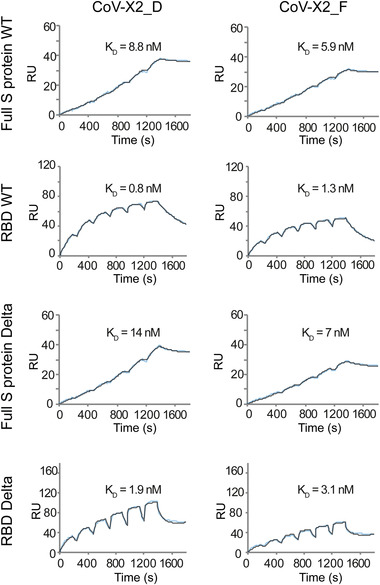
Characterization of the affinity‐matured bispecific antibodies by surface plasmon resonance. Binding of the affinity‐matured antibodies CoV‐X2_D and CoV‐X2_F was investigated by surface plasmon resonance. The antibodies were immobilized on the chip surface at 25 nm. Recombinant full S protein or receptor binding domain (RBD) preparations of either wild‐type (WT) or Delta variant SARS‐CoV‐2 were injected at increasing concentrations (1.56, 3.12, 6.25, 12.5, 25, and 50 nm). Calculated equilibrium dissociation constant (*K*_D_) values are reported in each plot.

**Figure 5:**
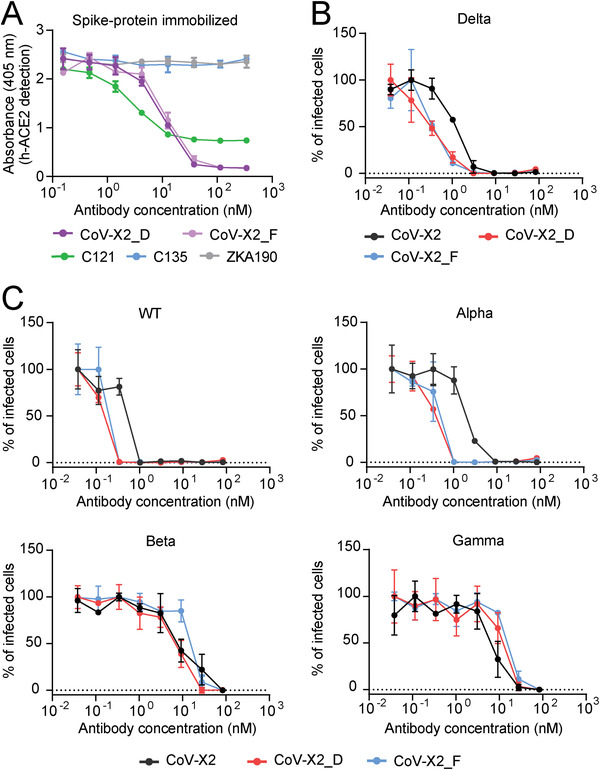
*In vitro* ACE2 inhibition and virus neutralization. A) The affinity‐matured bispecific antibodies CoV‐X2_D and CoV‐X2_F inhibited full S glycoprotein binding to human ACE2. ACE2 binding to antibody‐S‐trimer complexes was measured with increasing concentrations of the indicated antibody and constant ACE2. B) The bispecific antibodies CoV‐X2_D and CoV_X2_F neutralized SARS‐CoV‐2 Delta variant isolates more potently compared to the parental bispecific antibody CoV‐X2. Shown is the mean of three experiments with SD. C) The bispecific antibodies CoV‐X2_D and CoV‐X2_F potently neutralized all tested SARS‐CoV‐2 isolates (wild‐type, alpha, beta, and gamma SARS‐CoV‐2). Shown is the mean of three experiments with SD.

The ability of the novel affinity‐matured bispecific antibodies to inhibit the binding of recombinant ACE2 to the S glycoprotein trimer was investigated by ELISA. The C135 antibody, which is known not to inhibit S‐ACE2 binding, and an unrelated antibody (ZKA190) were used as negative controls.^[^ ^36^ ^]^ C121 alone, which covers the ACE2‐binding site on the RBD, prevented ACE2 binding only partially. In contrast, ACE2 binding was not detected when CoV‐X2_D and CoV‐X2_F were added, pointing to a synergistic effect by the two different arms that comprise the bispecific antibodies (**Figure** **5**A).

Virus neutralization was assessed by plaque‐reduction neutralization assays with infectious virus isolated from human donors. The affinity‐matured bispecific antibodies CoV‐X2_D and CoV‐X2_F displayed very high virus neutralization potency on all the variants tested, with improved half maximal effective concentration (EC_50_) and EC_90_ values for the Delta variant in respect to the parental CoV‐X2 (Figure 5B,C and **Table** **2**). Hence, neutralization improvement against the desired variant was achieved without causing a loss of potency in other variants.

**Table 2:** Quantification of virus neutralization (10^−9^ m)

Antibody	EC_50_ WT	EC_90_ WT	EC_50_ Delta variant	EC_90_ Delta variant
CoV‐X2	1.2	1.0	2.5	2.2
CoV‐X2_D	0.1	0.07	0.4	0.1
CoV‐X2_F	0.1	0.05	0.7	0.2

## 3 Discussion

Neutralizing mAbs have been successfully employed for prophylaxis and treatment of patients infected with SARS‐CoV‐2.^[^ ^26^, ^27^, ^28^, ^29^ ^]^ The emergence of immune escape variants, however, constantly challenges the efficacy of approved products.^[^ ^30^, ^33^, ^38^ ^]^ All SARS‐CoV‐2 antibodies described so far had a similar fate, including approved antibody cocktail products such as REGEN‐COV, Evusheld, Xevudy, and Regkirona, which had significantly diminished clinical activity in protecting patients from infections with recent SARS‐CoV‐2 variants.^[^ ^32^, ^39^, ^40^, ^41^ ^]^ A possible solution might involve the development of “universal antibodies” against conserved epitopes that are crucial to the survival of a given virus, but this strategy has so far failed even for long‐known pathogens such as influenza. In order to continue the effective use of antibody‐based therapies, it is important to constantly develop new molecules against the new variants. This is certainly true for a rapidly mutating virus such as SARS‐CoV‐2, but it also applies to virtually all pathogens with sequence variability developing over time or geographical distribution. Additionally, the prediction of future mutations, using artificial intelligence or purely randomized libraries, might help to speed up the development of antibodies capable of neutralizing novel variants.

Instead of discovering antibodies ex novo each time a new variant arises, it can be attractive to rapidly adapt existing antibodies to it. The process is somehow reminiscent of the commonly performed adaptation of influenza vaccines to new circulating viral strains, which allows relaxed regulatory requirements resulting in time‐ and cost‐saving.

Here we describe a methodology to adapt existing neutralizing antibodies of proven efficacy to emerging pathogen variants. The approach is based on a structure‐guided affinity maturation of existing antibodies using combinatorial mutagenesis and phage display technology. Rather than randomizing residues in all CDRs of the antibody, our procedure focuses on residues predicted to be relevant for interaction with the mutated antigen. This allows an extensive exploration of the conformational space around the mutated region while preserving the critical interactions in the conserved part of the epitope, thus avoiding the loss of binding and neutralization of other variants. The key to this approach is defining which antibody residues are likely to be important for interaction with the desired target. In our experience, it is better to avoid focusing strictly on antibody residues that, in experimental structures of the starting complex, are in direct contact with the antigen residues mutated in the pathogen variant. Proteins, and especially antibody CDR loops, can often alter their local structure to adapt to slightly different interfaces, such as a mutated epitope. Being too strict when defining the antibody residues to be randomized may not allow optimization of all the potentially available intermolecular interactions. A healthy degree of structural imagination, striking a balance between changing the entire CDRs, and being too narrow, is often more effective even than sophisticated computational simulations.

As a proof of concept, we applied the reported procedure to an existing neutralizing antibody. CoV‐X2 is a bispecific IgG‐like antibody that combines the specificity of two different mAbs, C121 and C135, isolated from patients recovered from an infection with the ancestral SARS‐CoV‐2 virus. CoV‐X2 is a very potent neutralizing antibody on different variants of concern, but it shows a reduced neutralization potency against the Delta variant. We combinatorially mutated amino acid residues in both binding specificities of CoV‐X2 based on structural data of the parental antibodies bound to the RBD of wild‐type SARS‐CoV‐2. The resulting affinity maturation libraries were subjected to an affinity capture procedure on recombinant Delta variant RBD. The binding specificities emerging from this procedure were deeply characterized *in vitro* and all isolated antibodies showed a remarkable similarity to the parental antibody in their biophysical characteristics. The best antibodies exhibited approximately sixfold (EC_50_) to twentyfold (EC_90_) improved virus neutralization against Delta, compared to the parental antibody, and no loss of binding or neutralization potency for other variants. It is difficult to pinpoint the relation between binding affinity and neutralization, especially when comparing molecules with high affinity and potency, and complex viral molecules such as the multimeric and highly dynamic spike protein. Nonetheless, the matured antibodies with improved neutralization show an increased association rate (from 3 × 10^6^ 1/Ms for the original antibody to 1 × 10^7^ for CoV‐X2_D and 1 × 10^7^ for CoV‐X2_F), suggesting that this might be relevant for the improved neutralization.

Interestingly, the best affinity matured antibodies, CoV‐X2_D and CoV‐X2_F, did not only display an improved neutralization potency against the Delta variant but also against the wild‐type virus. We postulate that this is a direct result and added bonus of the affinity maturation strategy described in this article. If we had only randomized the few antibody residues in direct, close contact with the mutated antigen residues in a specific X‐ray structure, as many might be tempted to do, we might not have explored a sufficiently large conformational space. Conversely, if we had randomized the entire CDR loop, we might have improved neutralization against Delta, but diminished neutralization/binding for other variants. Randomizing antibody residues in the vicinity of the mutated antigen, but not just strictly in contact with it, in order to account for structural plasticity, allowed optimization of the Delta interface, as planned, but also of the nearby wild‐type interactions. In our experience, it is critical to find a healthy balance between changing the entire CDRs and only mutating the very few antibody residues in close, direct contact with the antigen residues evolved in the pathogen variant.

The antibody optimization strategy described in this article relies on phage display technology. This methodology for discovering novel binding specificities or for evolving existing binders can be rapidly implemented, as library construction mainly relies on a combinatorial mutagenesis step, which can be performed by PCR, and on conventional cloning procedures in *E. coli*. The technology, however, crucially relies on the availability of good‐quality preparations of recombinant antigen. Ideally, the target protein of interest should be biotinylated at site‐specific positions, thus facilitating an immobilization on streptavidin‐coated solid support without loss of immunoreactivity. In our experience (ref. [42] and unpublished data), a single round of panning is adequate in affinity maturation campaigns to selectively isolate antibodies with enhanced binding kinetics, while maintaining a high degree of sequence diversity. In contrast, the de novo isolation of antibodies from phage display libraries typically requires two or more rounds of panning to attain an optimal balance between enrichment of high‐affinity binders and retaining a high degree of sequence diversity.^[^ ^43^, ^44^, ^45^, ^46^, ^47^ ^]^

In a pharmaceutical context, the facile isolation of antibody clones specific to viral variants risks being ineffective if the new antibodies cannot be rapidly produced as clinical‐grade material. The manufacturing process of antibody‐based products, following good manufacturing practice (GMP), is a tightly regulated process that typically takes one year from cell line development to the required threemonth stability data of the drug product. These timelines are hardly competitive with the rapid emergence of variants as the example of SARS‐CoV‐2 has shown. A possible avenue to accelerate the production of clinical grade material might be a temporarily relaxed interpretation of GMP guidelines by regulatory authorities, in the contingency of certain humanitarian crises, without compromising the quality of the product. This might be facilitated by the adaption of existing neutralizing antibodies that are remarkably similar to the original molecule rather than selecting antibodies ex novo.

## 4 Experimental Section

### Cell Lines

Chinese Hamster Ovary (CHO) cells and Vero E6 cells were obtained from the American Type Culture Collection (ATCC). Expi293F cells were obtained from ThermoFisher. Cell lines were expanded and stored as cryopreserved aliquots in liquid nitrogen. Cells were grown according to the manufacturer's instructions and kept in culture for no longer than 14 passages. Authentication of the cell lines including post‐freeze viability, growth properties, morphology, test for mycoplasma contamination, isoenzyme assay, and sterility tests were performed by the cell bank before shipment.

### Cloning, Expression, and Biotinylation of RBD WT and RBD Delta Variant

Codon‐optimized genes encoding residues 1–1208 of SARS‐CoV‐2 S ectodomain wild‐type (GenBank: MN908947) and Delta variant (GenBank: QWK65230.1) were synthesized and cloned into the mammalian expression vector pcDNA3.1(+) by Genscript; the sequences contained proline substitutions at residues 986 and 987 (S‐2P), a “GSAS” substitution at the furin cleavage site (residues 682–685), a C‐terminal T4 fibritin trimerization motif and a C‐terminal octa‐histidine tag. Plasmids for the production of RBD wild‐type (GenBank: MN908947, residues 319–537) and Delta variant (GenBank: QWK65230.1, residues 319–537) were similarly designed and obtained from Genscript. The gene strand featured a C‐terminal BirA target sequence (GLNDIFEAQKIEWHE), allowing for site specific biotinylation, as well as an octa‐histidine tag. The antigens were expressed in Expi293F cells by transient polyethyleneimine (PEI) transfection and purified by His‐Tag affinity chromatography after six days. Purified protein was analyzed by SDS‐PAGE under reducing and non‐reducing conditions, by SEC using a Superdex 75 increase on an Äkta Pure fast protein liquid chromatography (GE Healthcare, Amersham Biosciences) and DLS on a DynaPro NanoStar (Wyatt Technology, software Dynamics v.7.1.7.16). Site‐specific biotinylation of the protein was carried out using BirA enzyme in BirA buffer (10 mm Tris pH 7.5, 200 mm NaCl, 5 mm MgCl_2_) following the protocol described by Fairhead and colleagues.^[^ ^48^ ^]^

### Cloning and Expression of hACE2‐mFc

A deoxyribonucleic acid (DNA) construct of human ACE2 fused to the fragment crystallizable (Fc) region of mouse IgG at the C terminus (hACE2‐mFc) cloned into the mammalian expression vector pcDNA3.1(+) was purchased from Genscript. hACE2‐mFc was produced by transient PEI transfection in Expi293F cells and purified from the cell supernatant six days after transfection by HiTrap Protein A HP (Cytiva), analyzed by SDS–PAGE, DLS (DynaPro NanoStar Wyatt Technology, software Dynamics v.7.1.7.16), and binding assays. All protein batches underwent quality control and biophysical characterization to ensure functionality, stability, lack of aggregation, and batch‐to‐batch reproducibility.

### Generation of Affinity Maturation Libraries

Structure‐guided affinity maturation can facilitate the adaption of existing neutralizing mAbs to emerging viral variants. The procedure is illustrated in detail based on the adaption of the previously published neutralizing bispecific antibody CoV‐X2^[^ ^36^ ^]^ to the Delta variant of SARS‐CoV‐2.

As a first step, both binding specificities of CoV‐X2 (termed C121 and C135) were reformatted into a scFv format, connecting the V_H_ and V_L_ domains using a flexible Gly_4_SerGly_4_SerGly_4_ linker.^[^ ^37^ ^]^ The primers that were used are listed in Table S1, Supporting Information. Two affinity maturation libraries were generated by combinatorial mutagenesis of selected residues in the CDR loops of the antibodies. The amino acids to be mutated were chosen based on structural information of the antibodies bound to the RBD of wild‐type SARS‐CoV‐2.^[^ ^25^ ^]^ Based on a qualitative and visual inspection, CDR residues that were shown to be in close contact with amino acids mutated in the RBD of the Delta variant were selected. CDR residues with a ≤7Å distance from the RBD were chosen, including those capable of interacting with the mutated variant upon minor structural adjustments, establishing, for instance, polar intermolecular interactions. In the authors' experience, it was important to use judgment and evaluation of the entire structural context to select the residues to be mutated, rather than relying on very strict, quantitative interpretation of distances in a specific structure. Even ignoring the fact that the available experimental structural information may not be precise, X‐ray and similar structures rarely manage to account for the conformational plasticity of antibodies and antigens, especially in a highly dynamic protein such as the SARS‐CoV‐2 spike.

Four amino acids in the CDR2 of the heavy chain of C121 (S52, V54, S55, and G57) and three amino acids in the CDR3 of the light chain of C135 (Y225, N226, and S227) were chosen to be randomized (Figure 1), resulting in a theoretical library size of 1.6 × 10^5^ for the C121 antibody and 8 × 10^3^ for the C135 antibody, respectively. Randomization was achieved by PCR using partially degenerate primers. Therefore, two DNA fragments were obtained by PCR on the parental antibody using primer pairs a/b and e/f for C121, and a/d and e/f for C135, respectively (**Table** **1** and Figure S1, Supporting Information). After gel‐purification, the two segments were assembled by PCR and further amplified using primers a/f. The V_H_–V_L_ combinations were digested with NcoI/NotI restriction enzymes (New England Biolabs) and cloned into the NcoI/NotI‐digested phagemid vector pHEN1.^[^ ^49^ ^]^ The ligated plasmid DNA was purified on NucleoSpin Gel and PCR clean‐up columns (Macherey Nagel) and electroporated into fresh electrocompetent *E. coli* TG‐1 cells. Electrocompetent *E. coli* TG‐1 cells were prepared by washing the cells twice with 1 mm HEPES/5% glycerol and twice with 10% glycerol in water. After the washing steps, the *E. coli* TG‐1 cells were resuspended in 10% glycerol in water to a density of ≈2 × 10^11^ cells. The *E. coli* TG‐1 cells were transformed with plasmid DNA, spread on 2xYT‐agar plates supplemented with 100 µg mL^−1^ ampicillin (Applichem) and 1% glucose (VWR), and incubated at 30°C overnight. The transformation efficiency was assessed by plating a serial dilution of the transformed *E. coli* TG‐1 cells onto 2xYT‐agar plates (supplemented with 100 µg mL^−1^ ampicillin and 1% glucose), and counting the colonies for each dilution after an overnight incubation at 37°C. Successfully transformed *E. coli* TG‐1 cells were rescued with 2xYT‐10% glycerol from the plates on the next day. 50 mL 2xYT, supplemented with 100 µg mL^−1^ ampicillin, were inoculated with rescued cells (OD_600nm_ = 0.07). The cells were grown at 37°C (180 rpm) until an OD_600nm_ of 0.4 was reached, followed by co‐infection with VCS‐M13 Interference‐Resistant Helper Phage (Agilent, Santa Clara, CA). The infected cells were incubated at 30°C (180 rpm) overnight in the presence of 100 µg mL−1 ampicillin and 33 µg mL−1 kanamycin (Applichem) to allow for the production of phage. The next day, phage particles were precipitated from the bacterial supernatant, using 20% polyethylene glycol/2.5 m NaCl and stored at −20 °C until further use.

### Isolation of Affinity‐Matured Antibodies by Phage Display Technology

The generated phage display affinity maturation libraries were subjected to one round of panning using an affinity capture procedure on recombinant Delta variant RBD, following the protocol described by Viti et al.^[^ ^50^ ^]^ Briefly, 120 pmol of biotinylated RBD Delta were immobilized on 60 µL streptavidin‐coated beads (Invitrogen, M‐280). After blocking in 4% milk‐phosphate buffer saline (PBS), 800 µL of scFv displaying phage were added (≈10^12^ transforming units of phage per mL; no negative selection was performed prior to the positive selection) and incubated for 1 ½ h. Beads were washed six times with 0.1% Tween 20 in PBS and subsequently six times with PBS. Bound phage were eluted with triethylamine (Sigma) and used to infect fresh *E. coli* TG‐1. Individual colonies were inoculated in 2xYT media supplemented with 100 µg mL^−1^ ampicillin. Expression of scFv was induced by adding 1 mm isopropyl β‐d‐1‐thiogalactopyranoside (Applichem) and incubating at 30 °C overnight while shaking (200 rpm). Plates were spun down at 4000 g for 15 min. and supernatants containing soluble antibody fragments were collected. The resulting antibody fragments were screened by ELISA on immobilized RBD WT and Delta. Clones yielding positive ELISA signals for both RBD WT and Delta were sequenced and chosen for further characterization.

### Cloning, Expression, and Characterization of Affinity‐Matured Antibodies

The asymmetric heterodimeric IgG‐based bispecific antibodies produced in this study consisted of two different heavy and light chains. One of the major issues in the generation of such molecules is the random association of heavy and light chains.^[^ ^51^ ^]^ An elegant approach to enforce correct light chain association is represented by the CrossMab technology.^[^ ^52^ ^]^ This methodology is based on the crossover of the antibody domain within one Fab arm of the bispecific antibody.^[^ ^53^ ^]^ The combination of CrossMab technology with other approaches that enable the correct association of heavy chains such as knobs‐into‐holes technology, therefore, greatly facilitats the generation of IgG‐based bispecific antibodies with correctly paired chains.^[^ ^54^ ^]^ The antibodies obtained by phage display selections were cloned into the CrossMab format by amplifying the genes for V_H_ and V_L_ by PCR and cloning them into the mammalian expression vector pMM137 (developed in‐house), resulting in a crossover between C_H1_ and C_L_ in the C135 moiety (Figures S8 and S9, Supporting Information). Bispecific antibodies were produced by PEI induced transient gene expression in CHO cells, followed by purification to homogeneity by Protein A affinity chromatography. Quality control of the bispecific antibodies was performed by SDS‐PAGE under reducing and non‐reducing conditions and size exclusion chromatography (Superdex200 10/300GL, GE Healthcare).

### Surface Plasmon Resonance Analysis

We analyzed the binding properties of the generated antibodies at 25°C on a Biacore 8K instrument (Cytiva) using 10 mm HEPES pH 7.4, 150 mm NaCl, 3 mm ethylenediaminetetraacetic acid, and 0.005% Tween‐20 as running buffer. Antibodies were immobilized at 25 nm on the surface of CM5 chips (Cytiva) through standard amine coupling. Increasing concentrations of antigens (1.56, 3.12, 6.25, 12.5, 25, and 50 nm) were injected using a single‐cycle kinetics setting; analyte responses were corrected for unspecific binding and buffer responses. Curve fitting and data analysis were performed with the Biacore Insight Evaluation Software v.2.0.15.12933.

### ACE2 Inhibition Assay

ELISA assays were used to investigate the antibodies´ ability to inhibit the binding of RBD and full S protein to hACE2. For this purpose, 96‐well ELISA plates were coated at 4 °C with 158 nm RBD or 37 nm full S protein, washed and blocked with PBS containing 10% fetal calf serum (FCS). Antibodies were then added at different dilutions (starting from 340 nm and serially diluted 1:3) and incubated for 1 h at 25°C; after washing, hACE2–mouse Fc was added at a constant saturating concentration (160 nm) and left for 1 h at 25°C. After further washing, bound hACE2 was detected using standard protocols with goat anti‐mouse IgG coupled to alkaline phosphatase (dilution 1:500, SouthernBiotech). After addition of p‐Nitrophenyl Phosphate (PNPP) substrate, colorimetric changes in the ELISA plates were measured at 405 nm with the reader software Gen5 version 1.11.5 (BioTek Instruments). The data were analyzed with GraphPad Prism version 8.4.2.

### SARS‐CoV‐2 Virus‐Neutralization Assay

The neutralizing activity of the affinity‐matured bispecific antibodies CoV‐X2_D and CoV‐X2_F against wild‐type and Delta variant SARS‐CoV‐2 was investigated by plaque‐reduction neutralization tests following a previously reported protocol.^[^ ^36^ ^]^ In brief, 50 µL of antibody, starting from a concentration of 12 µg mL^−1^ in a serial threefold dilution, were mixed in a flat‐bottomed tissue culture microtiter plate (COSTAR) with an equal volume of 100 median tissue culture infectious dose of infectious virus that was isolated from patients with Coronavirus disease, sequenced, titrated, and incubated at 33°C in 5% CO2. After 1 h, 3 × 10^4^ (100 µL) Vero E6 cells (VERO C1008, Vero 76, clone 18 E6, Vero E6; ATCC CRL‐1586) were added to each well. After 3 days of incubation, the cells were stained with Gram's crystal violet solution (Merck) plus 5% formaldehyde 40% m/v (Carlo Erba S.p.A.) for 30 min. The microtiter plates were then washed in water and the wells were analyzed to evaluate the degree of cytopathic effect compared to the untreated control. Each experiment was performed in triplicate. The EC_50_ was determined using three‐parameter nonlinear regression (GraphPad Prism).

## Conflict of Interest

D.N. is a co‐founder and shareholder of Philogen, a Swiss‐Italian Biotech company that operates in the field of ligand‐based pharmacodelivery. F.P., L.P., A.E., M.M., and R.D.L. are employees of Philochem AG, daughter company of Philogen. The Institute for Research in Biomedicine has filed an International Patent Application No. PCT/EP2021/084708 on 8 December 2021 with the title “Multispecific Antibodies against Severe Acute Respiratory Syndrome Coronavirus 2” and published under No. “WO2022/122788.”

## Author Contributions

F.P. and M.P. contributed equally to this work. Conception and design: F.P., M.P., R.C., L.S., R.D.L., D.N., and L.V. Development of methodology: F.P., M.P., R.C., L.S., M.M., D.N., and L.V. Acquisition of data: F.P., M.P., L.S., R.C., R.D.G., E.T., L.P., A.E., M.M., I.C., and J.C.S. Analysis and interpretation of data: F.P., M.P., L.S., R.D.G., R.D.L., D.N., L.V., A.P., and F.B. Study supervision: R.D.L., D.N., and L.V.

## Supporting information

Supporting Information

## Data Availability

The data of this article are mostly shown in the supplementary information. Data not shown in the supplementary information can be obtained from the corresponding author upon reasonable request.
