# Supplementary material for: Adapting Neutralizing Antibodies to Viral Variants by Structure‐Guided Affinity Maturation Using Phage Display Technology
Source: Glob Chall. 2023 Aug 21;7(10):2300088. doi: 10.1002/gch2.202300088 (PMC10566804; doi:10.1002/gch2.202300088)
Supplement: Supplementary file 1 — Supporting Information [file GCH2-7-2300088-s001.pdf]

Global Challenges
Open Access

Supporting Information

for *Global Challenges.*, DOI 10.1002/gch2.202300088

Adapting Neutralizing Antibodies to Viral Variants by Structure-Guided Affinity Maturation Using Phage Display Technology

*Frederik Peissert, Mattia Pedotti, Riccardo Corbellari, Luca Simonelli, Raoul De Gasparo, Elia Tamagnini, Louis Plüss, Abdullah Elsayed, Mattia Matasci, Roberto De Luca, Irene Cassaniti, Jose' Camilla Sammartino, Antonio Piralla, Fausto Baldanti, Dario Neri and Luca Varani**

Supplementary Material

Adapting Neutralizing Antibodies to Viral Variants by Structure-Guided Affinity Maturation using Phage Display Technology

Frederik Peissert[a,1], Mattia Pedotti[a,2], Riccardo Corbellari[1], Luca Simonelli[2], Raoul De Gasparo[2], Elia Tamagnini[2], Louis Plüss[1], Abdullah Elsayed[1], Mattia Matasci[1], Roberto De Luca[1], Irene Cassaniti[4], Jose' Camilla Sammartino[4], Antonio Piralla[4], Fausto Baldanti[4,5] Dario Neri[1,3], and Luca Varani[2]

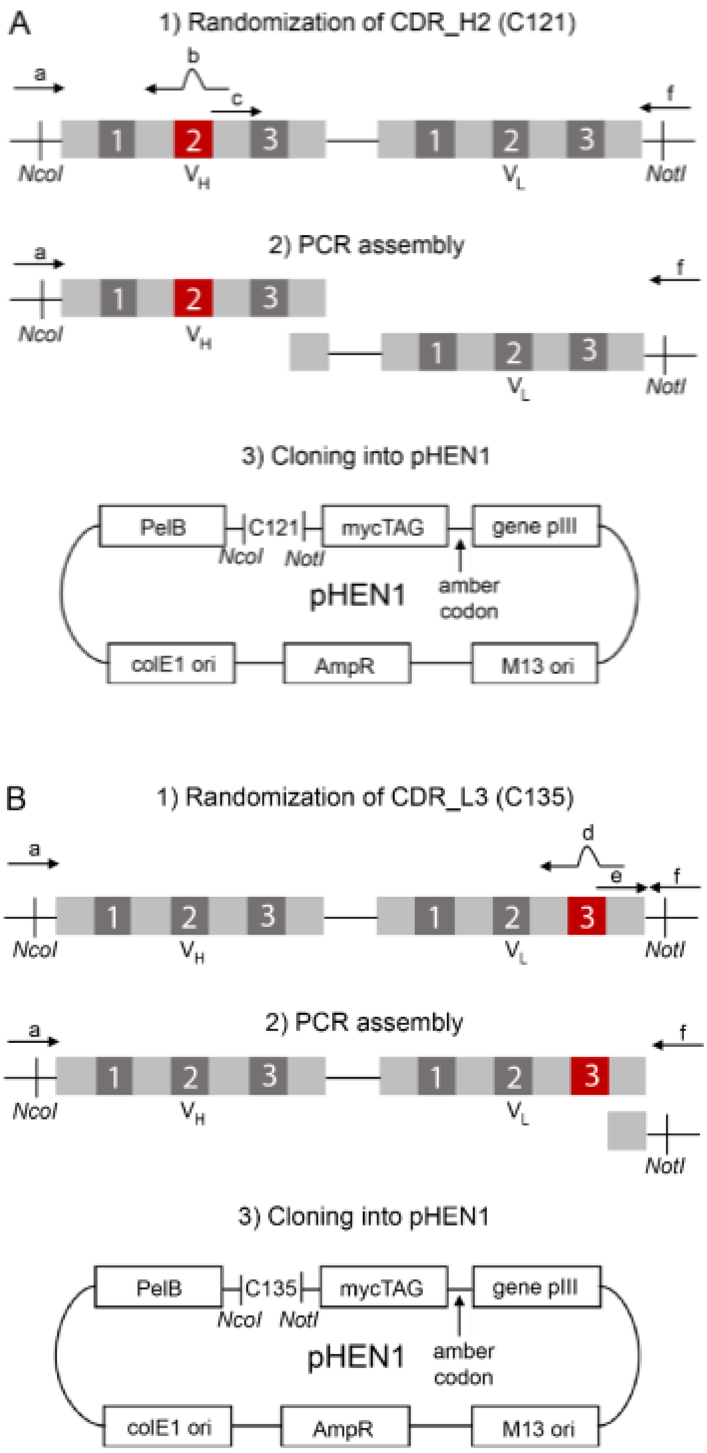

Supplementary Figure 1: Cloning of affinity-maturation libraries. The antibodies C121 (A) and C135 (B) were cloned into a scFv fragment linking the V_H and the V_L using a $G_4SG_4SG_4$ linker. For C121, amino acids in the CDR2 of V_H were randomized using the indicated partially degenerate primer (see **Table 1**). For C135, amino acids in the CDR3 of the V_L were randomized using the indicated partially degenerate primers (see **Table 1**). The resulting antibody libraries were cloned into the pHEN1 vector, using the *Nco*I and *Not*I restriction sites.

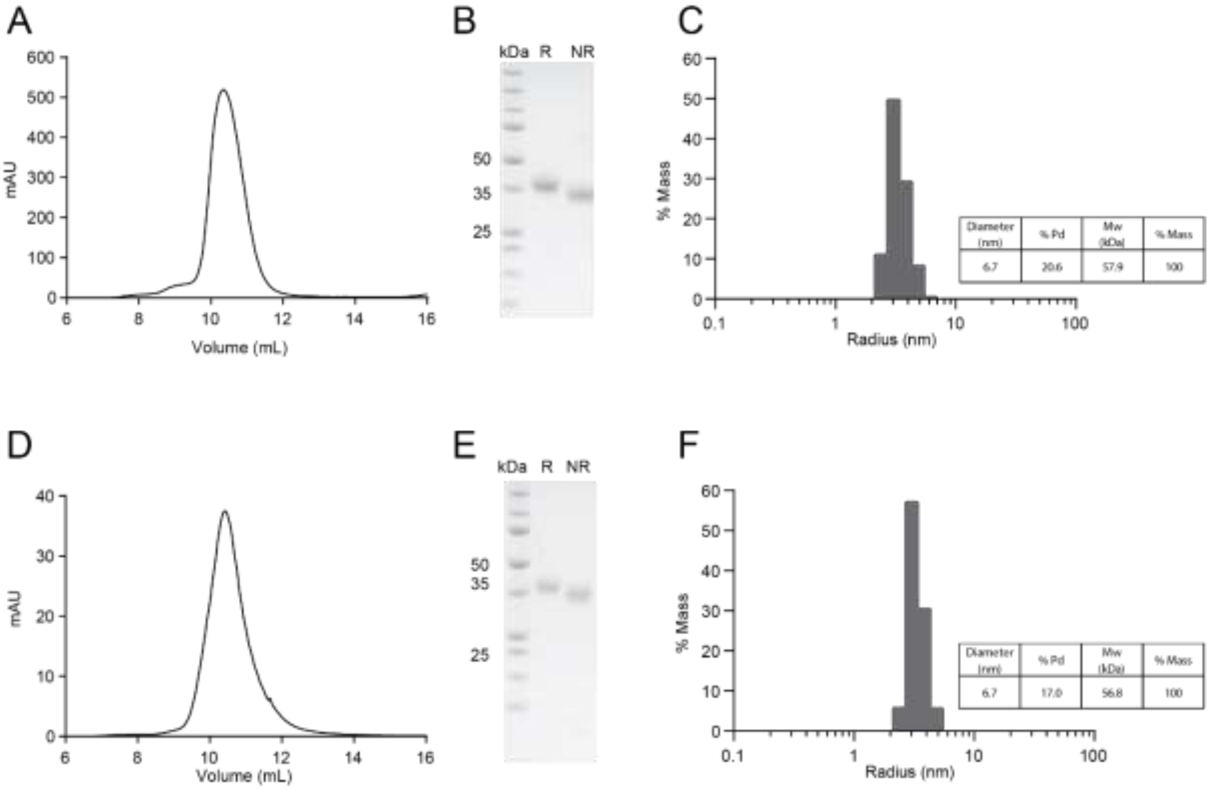

Supplementary Figure 2: Expression, and characterization of RBD wild-type and Delta variant. (A) SEC profile of RBD WT, showing a clean peak with no signs of aggregation. (B) SDS-PAGE confirmed the correct size of the recombinantly produced RBD WT. (C) Dynamic light scattering of RBD WT, indicating the monodispersity distribution of the sample with a unique peak corresponding to the molecular weight of the glycosylated RBD. (D) SEC profile of RBD Delta variant, showing a clean peak with no signs of aggregation. (E) SDS-PAGE confirmed the correct size of the recombinantly produced RBD Delta variant. (F) Dynamic light scattering of RBD Delta variant, indicating the monodispersity distribution of the sample with a unique peak corresponding to the molecular weight of the glycosylated RBD.

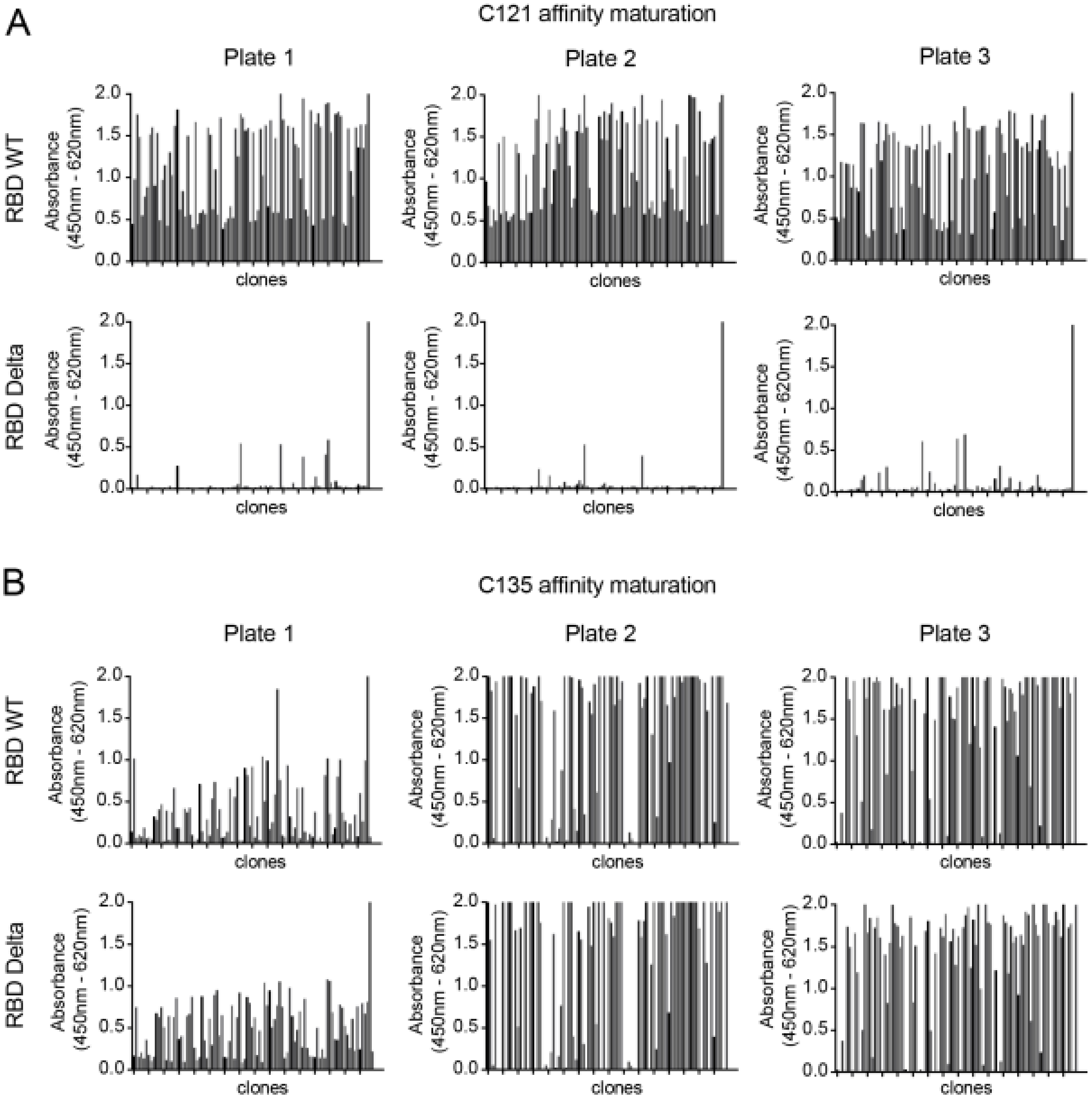

Supplementary Figure 3: ELISA results after phage display selections. Affinity-maturation libraries of C121 and C135 were screened in one round of biopanning against a recombinant preparation of the Delta variant RBD. Isolated clones were tested for binding to wild-type and Delta variant recombinant RBD by ELISA. Results of the phage display selection for C121 (A) and C135 (B).

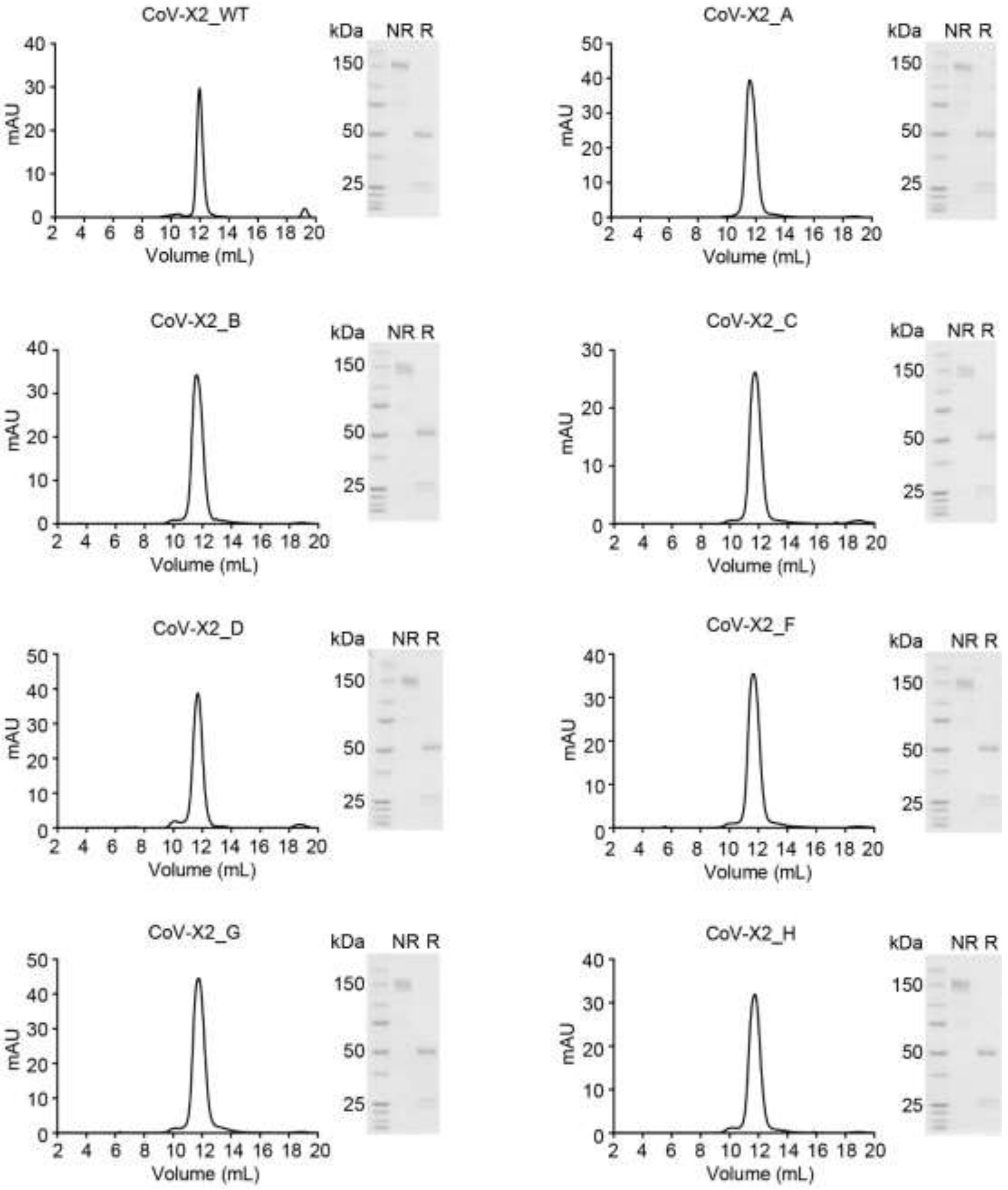

Supplementary Figure 4: Biochemical characterization of affinity-matured bispecific antibodies in CrossMab™ format. All bispecific antibodies showed a single peak in SEC with no signs of aggregation and a clean profile in SDS-PAGE with bands at expected size.

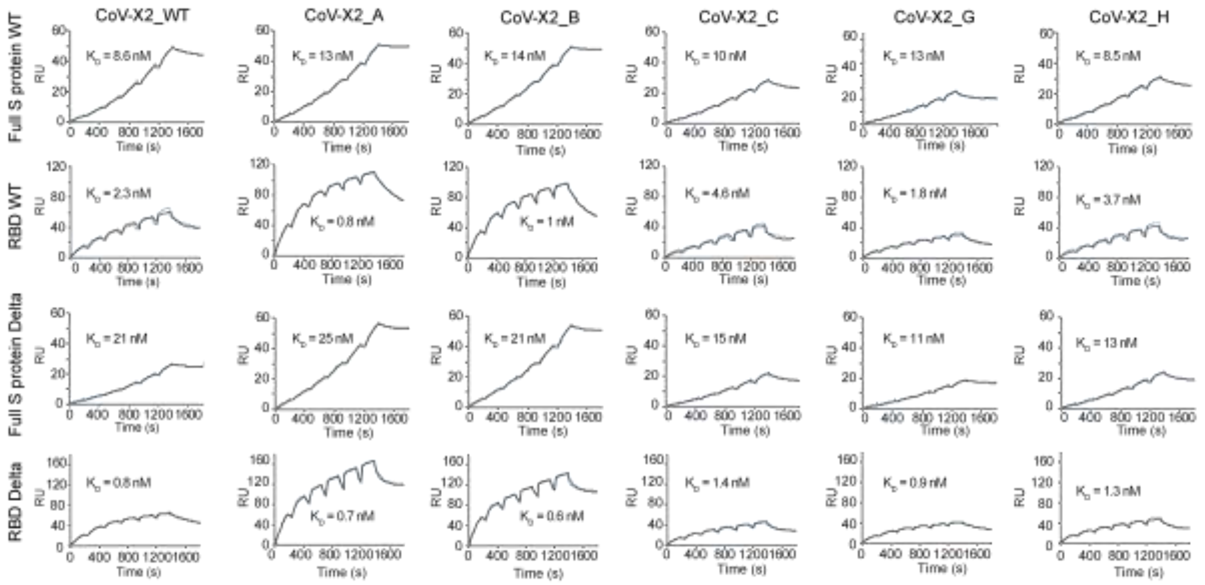

Supplementary Figure 5: Characterization of additional affinity-matured bispecific antibodies by surface plasmon resonance. Binding of the affinity-matured antibodies CoV-X2_A, CoV-X2_B, CoV-X2_C, CoV-X2_G and CoV-X2_H was investigated by surface plasmon resonance and compared to the affinity of the parental antibody (CoV-X2). Antibodies were immobilization on the chip surface at 25 nM. Recombinant full S protein or RBD preparations of either WT or Delta variant SARS-CoV-2 were injected at increasing concentrations (1.56, 3.12, 6.25, 12.5, 25, 50 nM). Calculated equilibrium dissociation constant (K_D) values are reported in each plot.

RVQPTESIVRFPNITNLCPFGEVFNATRFASVYAWNRKRISNCVADYSVLYNSASFST
FKCYGVSPTKLNDLCFTNVYADSFVIRGDEVRQIAPGQTGKIADYNYKLPDDFTGCV
IAWNSNNLDSKVGGNYNYLYRLFRKSNLKPFERDISTEIYQAGSTPCNGVEGFNCYF
PLQSYGFQPTNGVGYQPYRVVVLSFELLHAPATVCGPKKSTNLVKNKHHHHHHHH
GLNDIFEAQKIEWHE

Supplementary Figure 6: Amino acid sequence of the recombinant wild-type SARS-CoV-2 RBD construct. Sequence of the RBD is shown in black, the 8xHis-tag is highlighted in red and the Avi-tag sequence for site-specific biotinylation is highlighted in blue.

RVQPTESIVRFPNITNLCPFGEVFNATRFASVYAWNRKRISNCVADYSVLYNSASFST
FKCYGVSPTKLNDLCFTNVYADSFVIRGDEVRQIAPGQTGKIADYNYKLPDDFTGCV
IAWNSNNLDSKVGGNYNYRYRLFRKSNLKPFERDISTEIYQAGSKPCNGVEGFNCYF
PLQSYGFQPTNGVGYQPYRVVVLSFELLHAPATVCGPKKSTNLVKNKHHHHHHHH
GLNDIFEAQKIEWHE

Supplementary Figure 7: Amino acid sequence of the recombinant Delta variant SARS-CoV-2 RBD construct. Sequence of the RBD is shown in black, the 8xHis-tag is highlighted in red and the Avi-tag sequence for site-specific biotinylation is highlighted in blue.

QSALTQPASVSGSPGQSITISCTGTSSDVGSYNLVSWYQQHPGKAPKLMIYEGSKRPS GVSNRFSGSKSGNTASLTISGLQAEDEADYYCCSYAGSSTLVFGGGTKLTVLGQPKA NPTVTLFPPSSEELQANKATLVCLISDFYPGAVTVAWKADGSPVKAGVETTKPSKQS NNKYAASSYLSLTPEQWKSHRSYSCQVTHEGSTVEKTVAPTECSQVQLVQSGAEVK KPGASVKVSCKASGYTFTGYYMHWVRQAPGQGLEWMGWISPVSGNTNYAQKFQG RVTMTRDTSISTAYMELSRLRSDDTAVYYCARAPLFPTGVLAGDYYYYGMDVWGQ GTTVTVSSASTKGPSVFPLAPSSKSTSGGTAALGCLVKDYFPEPVTVSWNSGALTSG VHTFPAVLQSSGLYSLSSVVTVPSSSLGTQTYICNVNHKPSNTKVDKKVEPKSCDKT HTCPPCPAPELLGGPSVFLFPPKPKDTLMISRTPEVTCVVVDVSHEDPEVKFNWYVD GVEVHNAKTKPREEQYNSTYRVVSVLTVLHQDWLNGKEYKCKVSNKALPAPIEKTI SKAKGQPREPQVYTLPPCRDELTKNQVSLWCLVKGFYPSDIAVEWESNGQPENNYK TTPPVLDSDGSFFLYSKLTVDKSRWQQGNVFSCSVLHEALHSHYTQKSLSLSPGKDI QMTQSPSTLSASVGDRVTITCRASQSISNWLAWFQQKPGKAPKLLIYEASSLESGVPS RFSGSGSGTEFTLTISSLQPDDFATYYCQQYFEYPWTFGQGTKVEIKSSASTKGPSVFP LAPSSKSTSGGTAALGCLVKDYFPEPVTVSWNSGALTSGVHTFPAVLQSSGLYSLSS VVTVPSSSLGTQTYICNVNHKPSNTKVDKKVEPKSCQVQLVESGGGVVQPGRSLRLS CAASGFTFSSYAMHWVRQAPGKGLEWVAVIPFDGRNKYYADSVTGRFTISRDNSKN TLYLQMNSLRAEDTAVYYCASSSGYLFHSDYWGQGTLVTVSSASVAAPSVFIFPPSD EQLKSGTASVVCLLNNFYPREAKVQWKVDNALQSGNSQESVTEQDSKDSTYSLSST LTLSKADYEKHKVYACEVTHQGLSSPVTKSFNRGECDKTHTCPPCPAPELLGGPSVF LFPPKPKDTLMISRTPEVTCVVVDVSHEDPEVKFNWYVDGVEVHNAKTKPREEQYN STYRVVSVLTVLHQDWLNGKEYKCKVSNKALPAPIEKTISKAKGQPREPQVCTLPPS RDELTKNQVSLSCAVKGFYPSDIAVEWESNGQPENNYKTTPPVLDSDGSFFLVSKLT VDKSRWQQGNVFSCSVLHEALHSHYTQKSLSLSPGK

Supplementary Figure 8: Amino acid sequence of the affinity matured bispecific antibody CoV-X2_D in CrossMab™ format. The C121 antibody domains (hole) are annotated as following: VL(C121), CL(C121), VH(C121), CH1(C121), Hinge(C121), CH2(C121), CH3(hole, C121). The C135 antibody domains (knob) are annotated as following: VL(C135), CH1(C135), Hinge(partial, C135), VH(C135), CL(C135), Hinge(partial, C135), CH2(C135), CH3(knob, C135). Amino acids that were subject to combinatorial mutagenesis during affinity maturation procedures are indicated in red.

QSALTQPASVSGSPGQSITISCTGTSSDVGSYNLVSWYQQHPGKAPKLMIYEGSKRPS
GVSNRFSGSKSGNTASLTISGLQAEDEADYYCCSYAGSSTLVFGGGTKLTVLGQPKA
NPTVTLFPPSSEELQANKATLVCLISDFYPGAVTVAWKADGSPVKAGVETTKPSKQS
NNKYAASSYLSLTPEQWKSHRSYSCQVTHEGSTVEKTVAPTECSQVQLVQSGAEVK
KPGASVKVSCKASGYTFTGYYMHWVRQAPGQGLEWMGWISPISGNTNYAQKFQGR
VTMTRDTSISTAYMELSRLRSDDTAVYYCARAPLFPTGVLAGDYYYYGMDVWGQG
TTVTVSSASTKGPSVFPLAPSSKSTSGGTAALGCLVKDYFPEPVTVSWNSGALTSGV
HTFPAVLQSSGLYSLSSVVTVPSSSLGTQTYICNVNHKPSNTKVDKKVEPKSCDKTHT
CPPCPAPELLGGPSVFLFPPKPKDTLMISRTPEVTCVVVDVSHEDPEVKFNWYVDGV
EVHNAKTKPREEQYNSTYRVVSVLTVLHQDWLNGKEYKCKVSNKALPAPIEKTISK
AKGQPREPQVYTLPPCRDELTKNQVSLWCLVKGFYPSDIAVEWESNGQPENNYKTT
PPVLDSDGSFFLYSKLTVDKSRWQQGNVFSCSVLHEALHSHYTQKSLSLSPGKDIQM
TQSPSTLSASVGDRVTITCRASQSISNWLAWFQQKPGKAPKLLIYEASSLESGVPSRFS
GSGSGTEFTLTISSLQPDDFATYYCQQYFEYPWTFGQGTKVEIKSSASTKGPSVFPLAP
SSKSTSGGTAALGCLVKDYFPEPVTVSWNSGALTSGVHTFPAVLQSSGLYSLSSVVT
VPSSSLGTQTYICNVNHKPSNTKVDKKVEPKSCQVQLVESGGGVVQPGRSLRLSCAA
SGFTFSSYAMHWVRQAPGKGLEWVAVIPFDGRNKYYADSVTGRFTISRDNSKNTLY
LQMNSLRAEDTAVYYCASSSGYLFHSDYWGQGTLVTVSSASVAAPSVFIFPPSDEQL
KSGTASVVCLLNNFYPREAKVQWKVDNALQSGNSQESVTEQDSKDSTYSLSSTLTLS
KADYEKHKVYACEVTHQGLSSPVTKSFNRGECDKTHTCPPCPAPELLGGPSVFLFPP
KPKDTLMISRTPEVTCVVVDVSHEDPEVKFNWYVDGVEVHNAKTKPREEQYNSTYR
VVSVLTVLHQDWLNGKEYKCKVSNKALPAPIEKTISKAKGQPREPQVCTLPPSRDEL
TKNQVSLSCAVKGFYPSDIAVEWESNGQPENNYKTTPPVLDSDGSFFLVSKLTVDKS
RWQQGNVFSCSVLHEALHSHYTQKSLSLSPGK

Supplementary Figure 9: Amino acid sequence of the affinity matured bispecific antibody CoV-X2_F in CrossMab™ format. The C121 antibody domains (hole) are annotated as following: VL(C121), CL(C121), VH(C121), CH1(C121), Hinge(C121), CH2(C121), CH3(hole, C121). The C135 antibody domains (knob) are annotated as following: VL(C135), CH1(C135), Hinge(partial, C135), VH(C135), CL(C135), Hinge(partial, C135), CH2(C135), CH3(knob, C135). Amino acids that were subject to combinatorial mutagenesis during affinity maturation procedures are indicated in red.

Supplementary Table 1: Primers for the cloning of C121 & C135 into scFv format

Name	Sequence
LMB3long	5’ CAG GAA ACA GCT ATG ACC ATG ATT AC 3’
C121_rev	5’ GAG TTT TTG TTC TGC GGC CGC CCG GCC TAG GAC GGT CAG CTT GG 3’
C121_SP_fwd	5’ TTG CCT ACG GCA GCC GCT GGA TTG TTA TTA CTC GCG GCC CAG CCG GCC ATG GCC CAG GTG CAG CTG GTG CAG TC 3’
C135_rev	5’ GAG TTT TTG TTC TGC GGC CGC CCG TTT GAT TTC CAC CTT GGT CC 3’
C135_SP_fwd	5’ TTG CCT ACG GCA GCC GCT GGA TTG TTA TTA CTC GCG GCC CAG CCG GCC ATG GCC CAG GTG CAG CTG GTG GAG TC 3’
P3_fwd	5’ GGG GCC GCA AGT GGT GGA GGC GGT TCA TAG ACT GTT GAA AGT TGT TTA GC 3’
pHEN1_BamHI_rev	5’ AAT GGA TCC TCA TTA AAG CCA GAA TGG AAA G 3’
MYC_fwd	5’ CGG GCG GCC GCA GAA CAA AAA CTC ATC TCA GAA GAG GAT CTG AAT GGG GCC GCA AGT GGT GGA GGC 3’
End_of_VL_C135_MycTag_fwd	5’ GGA CCA AGG TGG AAA TCA AAC GGG CGG CCG CAG AAC AAA AAC TC 3’
End_of_VL_C121_MycTag_fwd	5’ CCA AGC TGA CCG TCC TAG GCC GGG CGG CCG CAG AAC AAA AAC TC 3’
HindIII_PelB_fwd	5’ CGC CAA GCT TGC ATG CAA ATT CTA TTT CAA GGA GAC AGT CAT AAT GAA ATA CCT ATT GCC TAC GGC AGC CGC TGG 3’
fdseqlong	5’ GAC GTT AGT AAA TGA ATT TTC TGT ATG AGG 3’

Supplementary Table 2: Calculated equilibrium dissociation constant (K_D) of the bispecific antibodies (10^{-9} M).

**Antibody**	WT full S	Delta full S	WT RBD	Delta RBD
CoV-X2	8.6	21	2.3	0.8
CoV-X2_A	13	25	0.8	0.7
CoV-X2_B	14	21	1	0.6
CoV-X2_C	10	15	4.6	1.4
CoV-X2_D	8.8	14	0.8	1.9
CoV-X2_F	5.9	7	1.3	3.1
CoV-X2_G	13	11	1.8	0.9
CoV-X2_H	8.5	13	3.7	1.3

Supplementary Table 3: Kinetics parameters of the bispecific antibodies.

Antibody	WT full S protein			Delta full S protein			WT RBD			Delta RBD		
	K_{on} (1/Ms)	K_{off} (1/s)	K_D (nM)	K_{on} (1/Ms)	K_{off} (1/s)	K_D (nM)	K_{on} (1/Ms)	K_{off} (1/s)	K_D (nM)	K_{on} (1/Ms)	K_{off} (1/s)	K_D (nM)
**CoV-X2**	1.32e+5	1.13e-3	8.6	9.23e+4	1.97e-3	21	2.46e+6	5.69e-3	2.3	2.85e+6	2.27e-3	0.8
**CoV-X2_A**	1.05e+5	1.40e-3	13	8.52e+4	2.12e-3	25	2.88e+6	2.19e-3	0.8	1.91e+7	1.42e-2	0.7
**CoV-X2_B**	1.17e+5	1.61e-3	14	9.49e+4	2.04e-3	21	3.43e+6	3.43e-3	1	2.10e+7	1.20e-2	0.6
**CoV-X2_C**	2.65e+5	2.73e-3	10	2.47e+5	3.74e-3	15	3.56e+6	1.62e-2	4.6	5.38e+6	7.32e-3	1.4
**CoV-X2_D**	1.72e+5	1.52e-3	8.8	1.63e+5	2.35e-3	14	2.73e+6	2.33e-3	0.8	1.24e+7	2.32e-2	1.9
**CoV-X2_F**	3.46e+5	2.06e-3	5.9	3.53e+5	2.65e-3	7	3.00e+6	3.86e-3	1.3	2.64e+7	8.22e-2	3.1
**CoV-X2_G**	2.74e+5	3.70e-3	13	2.29e+5	2.54e-3	11	2.71e+6	4.95e-3	1.8	3.30e+6	3.00e-3	0.9
**CoV-X2_H**	3.45e+5	2.94e-3	8.5	2.99e+5	3.78e-3	13	3.23e+6	1.18e-2	3.7	4.61e+6	6.11e-3	1.3
